# *PIN1* promoter polymorphism (−842 G>C) contributes to a decreased risk of cancer: Evidence from meta-analysis

**DOI:** 10.3892/ol.2014.2280

**Published:** 2014-06-24

**Authors:** LIANG-JUN TAO, YI-SHENG CHEN, LEI YAO, BIN ZOU, LING-SONG TAO, JIAN KONG, YING-QING LIU, QIANG CAO, CHANG-JUN YIN

**Affiliations:** 1 Department of Urology and Institute of Prostatic Diseases, The Affiliated Wuhu No. 2 People’s Hospital of Wannan Medical College, Wuhu, Anhui 241000, P.R. China; 2 Department of Neurosurgery, The First Affiliated Hospital of Nanjing Medical University, Nanjing, Jiangsu 210029, P.R. China; 3 State Key Laboratory of Reproductive Medicine, Department of Urology, The First Affiliated Hospital of Nanjing Medical University, Nanjing, Jiangsu 210029, P.R. China

**Keywords:** *PIN1*, polymorphism, cancer, meta-analysis

## Abstract

Peptidyl-prolylcis-trans isomerase NIMA-interacting 1 (encoded by the *PIN1* gene) regulates the conformation of proline-directed phosphorylation sites and is important in the etiology of cancer. Since the identification of a functional polymorphism of *PIN1, (*−842 G>C; rs2233678), in the *PIN1* promoter region, numerous studies have evaluated the association between the *PIN1* promoter polymorphism (−842 G>C) and cancer risk. However, the available results are inconclusive. To derive a more precise estimation, a meta-analysis of seven previous case-control studies was performed, which included 4,524 cases exhibiting different tumor types and 4,561 control subjects. The published literature was retrieved from PubMed and EMBASE. Crude odds ratios (ORs) with 95% confidence intervals (CIs) were calculated to evaluate the strength of the association. Overall, the results of the present study demonstrated that individuals carrying the variant C allele (G/C and C/C) were associated with a significantly decreased cancer risk (OR, 0.75; 95% CI, 0.62–0.90 for GC vs. GG; OR, 0.75; 95% CI, 0.64–0.88 for GC/CC vs. GG). In further stratified analyses, a decreased cancer risk was observed in the following subgroups: Breast and lung cancer patients, Asian individuals, and in studies with a sample size >500. The results indicated that the *PIN1* promoter polymorphism (−842 G>C; rs2233678) contributes to a decreased risk of cancer via attenuating the transcriptional activity.

## Introduction

Cancer is a multifactorial disease that results from complex interactions between numerous genetic and environmental factors (1). Accumulative etiologic factors associated with cancer have been well-established by epidemiological studies, including alcohol consumption, cigarette smoking, obesity, occupational exposures, family history of cancer and diet. However, only a marginal number of the individuals that are exposed to these factors develop cancer, therefore, this indicates that genetic susceptibility is a more significant indication of an individual’s risk of cancer.

Proline (Pro)-directed phosphorylation, also termed phosphorylation of proteins on serine (Ser) or threonine (Thr) residues, is a critical intracellular signaling mechanism, which regulates diverse cellular processes, including cell cycle progression, transcriptional regulation, RNA processing, and cell proliferation and differentiation (2,3). It has been demonstrated that the deregulation of Pro-directed phosphorylation is a prevalent and specific event in human cancers, which results in cell transformation and oncogenesis (2).

Peptidyl-prolyl cis-trans isomerase NIMA-interacting 1 (encoded by the *PIN1* gene) specifically isomerizes the conformation of Pro-directed phosphorylation sites, revealing a novel post-phosphorylation regulatory mechanism (4,5). *PIN1* exhibits a high specificity for substrates with Ser/Thr-Pro motifs, and changes the conformation of phosphoproteins by recognizing and binding to specific phospho-Ser/Thr-Pro motifs (6). *PIN1* substrates, which contain phospho-Ser/Thr-Pro motifs, include a number of important cell cycle regulators, as well as oncogenic and tumor suppressor proteins. These include cyclin D1 (7), p53 (8), Cdc25 (9), c-Myc (10), c-Jun (8), β-catenin (11), glycogen synthase kinase-3β (10) and Bcl-2 (12). Therefore, by targeting these significant substrates, which contain phospho-Ser/Thr-Pro motifs, *PIN1*-induced conformational changes may function as a critical catalyst for the potentiation of multiple oncogenic signaling pathways during cancer development (13). It has been reported that *PIN1* is aberrantly overexpressed in numerous types of cancer, including prostate and lung cancer (14), esophageal squamous cell carcinoma (7), and breast cancer (8,11). By contrast, the inhibition of *PIN1* in cancer cells may trigger apoptosis or suppress the transformed phenotype (15,16). These results indicated that the *PIN1* gene may exhibit an oncogenic role in tumorigenesis.

The human *PIN1* gene, which is located at chromosome 19p13, contains four exons within a 14-kb region, encodes a 163-amino-acid protein and has a promoter region of 1.5 kb (17). Several putative functional single nucleotide polymorphisms (SNPs) have been identified in the coding and promoter regions of *PIN1*, and the most important is rs2233678 G>C: c.−842 G>C (842 nucleotides upstream of the initiation transcription code ATG) in the promoter. The *PIN1* promoter polymorphism (−842 G>C) was first identified in a study of Alzheimer’s disease, which revealed that the variant −842C allele was associated with an increased risk of Alzheimer’s disease (18). Recent studies have investigated the association between the *PIN1* promoter polymorphism (−842 G>C) and the risk of cancer in various organs, including the liver (19), lungs (17) and breast (20,21), squamous cell carcinoma of the head and neck (22), and nasopharyngeal (23) and esophageal cancer (24). However, the results of these studies remain controversial. Considering the extensive role of *PIN1* in the carcinogenic process, a meta-analysis was performed that included all eligible case-control studies, to estimate the overall cancer risk associated with the *PIN1* promoter polymorphism (−842 G>C) and to quantify the potential heterogeneity between the studies.

## Methods

### Identification and eligibility of relevant studies

All available case-control studies regarding the association between the *PIN1* promoter polymorphism (−842 G>C) and cancer risk were included in the present study. PubMed (www.ncbi.nlm.nih.gov/pubmed) and EMBASE (www.elsevier.com/online-tools/embase) were searched using the terms ‘*PIN1*’, ‘polymorphism’ and ‘cancer’ (last accessed: 8 May 2013). The search was limited to studies written in English. Additional studies were identified by manually searching the references of the original studies identified using the search terms. When more than one study investigating the same type of population was included in several publications, the most recent studies with the largest sample sizes were selected. Furthermore, studies included in the meta-analysis had to meet the following inclusion criteria: i) Evaluation of the *PIN1* promoter polymorphism (−842 G>C) and the cancer risk; ii) use of a case-control design; and iii) contained an available genotype frequency.

### Data extraction

Two authors independently extracted data from the studies according to the inclusion criteria and subsequently reached a consensus on the data items. The following characteristics were recorded from each study: The first author’s surname, year of publication, cancer type, country of origin, patient ethnicity, source of the control groups (population- or hospital-based controls), genotyping method, the number of cases and controls, and the P-value for the Hardy-Weinberg equilibrium (HWE) for the controls (Table I). Ethnicity was categorized as European or Asian and when studies included subjects from different ethnic groups, the data were extracted separately for each ethnic group where possible. Studies investigating more than one sample were included as individual data sets.

### Statistical analysis

For the control group of each study, the allelic frequency was calculated, and HWE was assessed using the χ^2^ test. P<0.05 was considered to indicate a statistically significant difference. The strength of the association between the *PIN1* promoter polymorphism (−842 G>C) and cancer risk was measured using odds ratios (ORs) with 95% confidence intervals (CIs). Firstly, the risks of the GC and CC genotypes for cancers were estimated, compared with the wild-type GG homozygote, and then the risks of GC/CC vs. GG and CC vs. GC/GG for cancers were evaluated, assuming dominant and recessive effects of the variant C allele, respectively. Stratified analyses were also performed by cancer type (when one cancer type was included in only one individual study, it was combined into the ‘other cancers’ group), ethnicity and sample size (populations >500 in the case and control groups). To investigate the potential for heterogeneity across the studies, a statistical test for heterogeneity was performed based on the Q-statistic (25) whereby P>0.1 indicated a lack of heterogeneity between the studies. The summary ORs estimate of each study was calculated using the fixed-effects model (the Mantel–Haenszel method) (26). Otherwise, the random-effects model (the DerSimonian and Laird method) (27) was used. Furthermore, the meta-regression model was used to investigate the possible source of heterogeneity among the different study types (28). Sensitivity analyses were performed to assess the stability of the results, whereby a single study in the meta-analysis was deleted each time to reflect the influence of the individual data set on the pooled OR. Publication bias was evaluated using a Begg’s funnel plot and Egger’s test (29). All analyses were performed using Stata software version 11.0 (StataCorp LP, College Station, TX, USA) and all tests were two-sided.

## Results

### Features of the studies

A total of seven articles in English regarding the *PIN1* promoter polymorphism (−842 G>C) and cancer risk were available for the present meta-analysis. One article investigated two individual samples, which were collected during different time periods. Another article investigated three individual samples that were obtained from different countries and each sample was counted as an individual study. Finally, a total of 10 case-control studies met the inclusion criteria, including 4,524 cases and 4,561 controls. The features of the selected studies are presented in Table I. All studies were case-control studies, including four breast cancer studies, two lung cancer studies, with the others categorized into an ‘other cancer’ group. There were three studies that included patients of European descent and seven studies including patients of Asian descent. The cancers were identified histologically or pathologically in the majority of studies and the polymerase chain reaction (PCR)-restriction fragment length polymorphism (RFLP) genotyping method was used. The distribution of genotypes in the controls of all studies was consistent with the HWE, with the exception of one study, which did not include data for the CC, GC and GG genotypes (19).

### Quantitative synthesis

**Figure 1:**
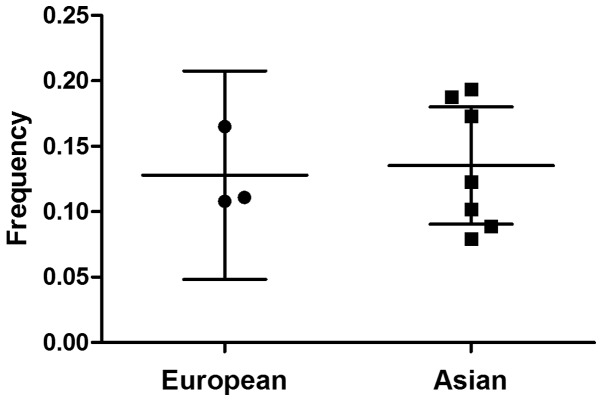
Peptidyl-prolyl cis-trans isomerase NIMA-interacting 1 C allele frequency between the control subjects stratified by ethnicity.

**Figure 2:**
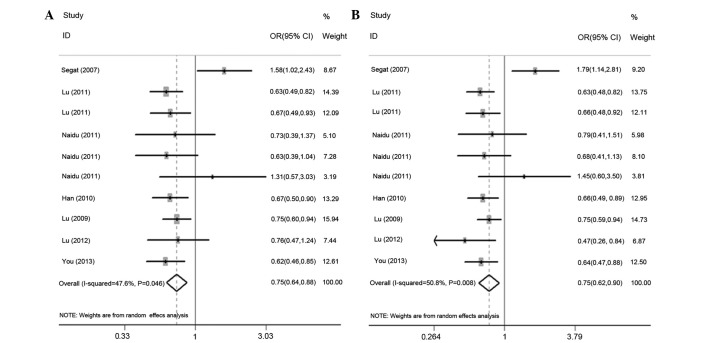
Forest plot of cancer risk associated with the *PIN1* promoter polymorphism (−842 G>C). (A) GC/CC vs. GG. (B) GC vs. GG. I^2^ quantifies the degree of heterogeneity in the meta-analysis. The squares and horizontal lines correspond to the study-specific OR and 95% CI. The area of each square reflects the study-specific weight (inverse of the variance). The diamond presents the summary OR and 95% CI. CI, confidence interval; OR, odds ratio.

A variation in the C allele frequency was identified across the different ethnicities. The mean C allele frequencies in the European and Asian populations were 12.8 and 13.5%, respectively (Fig. 1). As shown in Table II, the variant genotypes were associated with a significantly decreased cancer risk in the dominant genetic model (OR, 0.75; 95% CI, 0.64–0.88; P_heterogeneity_ =0.046; Fig. 2A). In addition, the variant GC heterozygote was associated with a decreased cancer risk when compared with the wild-type homozygote GG (OR, 0.75; 95% CI, 0.62–0.90; P_heterogeneiy_ =0.008; Fig. 2B). Furthermore, in the stratified analysis, significantly decreased risks of breast cancer (OR, 0.72; 95% CI, 0.57–0.90; P_heterogeneity_ =0.408 for GC vs. GG; OR, 0.71; 95% CI, 0.56–0.88; P_heterogeneity_ =0.493 for GC/CC vs. GG genotype) and lung cancer (OR, 0.64; 95% CI, 0.52–0.79; P_heterogeneity_ =0.814 for GC vs. GG; OR, 0.65; 95% CI, 0.53–0.79, P_heterogeneity_ =0.762 for GC/CC vs. GG genotype) were identified. In the subgroup analysis of ethnicity, a significantly decreased risk of cancer was identified among Asians for the GC vs. GG genotype (OR, 0.66; 95% CI, 0.57–0.76; P_heterogeneity_ =0.564) and the GC/CC vs. GG genotype (OR, 0.67; 95% CI, 0.58–0.77; P_heterogeneity_ =0.777). According to the sample size, a markedly decreased risk was identified in the subgroup with a sample size >500 (OR, 0.68; 95% CI, 0.59–0.78; P_heterogeneity_ =0.786 for GC vs. GG; OR, 0.68; 95% CI, 0.59–0.78; P_heterogeneity_ =0.738 for the GC/CC vs. GG genotype).

### Test of heterogeneity

Significant heterogeneity was identified in the heterozygote comparison (GC vs. GG, P_heterogeneity_ =0.008) and dominant model comparison (GC/CC vs. GG, P_heterogeneity_ =0.046), although not in the homozygote comparison (CC vs. GG, P_heterogeneity_ =0.273) or the recessive model comparison (CC vs. GC/GG, P_heterogeneity_ =0.181). The source of heterogeneity was also assessed in the heterozygote comparison by the year of publication, cancer type (breast, lung, and other types of cancer), ethnicity (European or Asian), and sample size (>500 subjects in each of the cases and control groups or ≤500). The year of publication (P=0.090) was found to contribute to substantial heterogeneity, however, no significant differences in cancer type (P=0.539), ethnicity (P=0.341) or sample size (P=0.434) were identified.

### Sensitivity analysis

**Figure 3:**
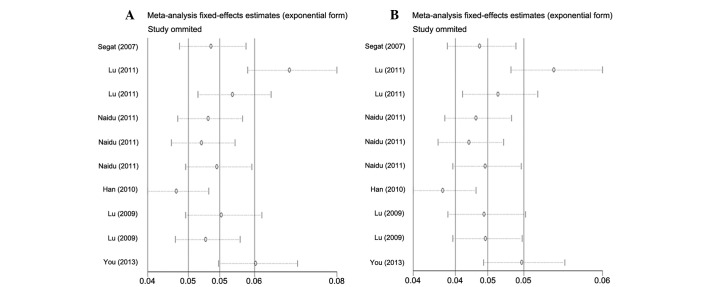
Sensitivity analysis of cancer risk associated with the *PIN1* promoter polymorphism (−842G>C). (A) GC/CC vs. GG. (B) GC vs. GG. The figure demonstrates the influence of individual studies on the summary odds ratio.

The influence of each study on the pooled OR was examined by repetition of the sensitivity analysis. As shown in Fig. 3, the sensitivity analysis indicated that the results of the present study were reliable and robust. Furthermore, when excluding the study by Segat *et al* (19), which did not contained the available data required for HWE, the estimated pool OR remained unchanged.

### Publication bias

**Figure 4:**
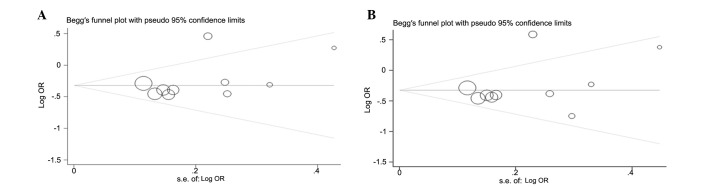
Begg’s funnel plot of publication bias. (A) GC/CC vs. GG. (B) GC vs. GG. Each point represents a separate study for the indicated association. Log (OR), natural logarithm of OR; Horizontal line, mean effect size; OR, odds ratio; s.e., standard error.

Begger’s funnel plot and Egger’s tests were used to assess the publication bias of the included studies. The graphical funnel plots appeared to be symmetrical in the dominant model comparison (Fig. 4A) and heterozygote comparison (Fig. 4B). Egger’s test was then used to provide statistical evidence of the funnel plot symmetry. As expected, the results did not reveal any evidence of publication bias (t=0.95 and P=0.370 for GC vs. GG; t=1.31 and P=0.228 for GC/CC vs. GG).

## Discussion

The present meta-analysis, which included 4,524 cases and 4,561 controls from seven case-control studies, evaluated the association between the *PIN1* promoter polymorphism (−842 G>C) and cancer risk. To the best of our knowledge, this is the first meta-analysis investigating this association. It was found that individuals exhibiting the variant GC and GC/CC genotypes were significantly associated with a decreased risk of cancer, particularly breast and lung cancer, which was revealed by the subgroup analysis. Furthermore, when stratifying the ethnicity and sample size, in Asian populations and sample sizes of >500, individuals with variant GC and GC/CC genotypes were also found to exhibit a decreased cancer risk.

*PIN1* is not an oncogene itself, however, it may present as an indispensable translator and amplifier of oncogenic signal transduction. *PIN1* specifically recognizes phospho-Ser/Thr-Pro motifs and regulates the conformation of Pro-directed phosphorylation sites, which potentiates multiple oncogenic signaling pathways during carcinogenesis (13). The overexpression of *PIN1* is a prevalent and specific event in human cancers (30). Consistently, studies have found that high *PIN1* expression correlates with poor prognosis in patients with different types of cancer, including prostate cancer (31) and esophageal squamous cell carcinoma (32). Thus, it is biologically possible that the functional polymorphisms of *PIN1* may be important in the etiology of cancer. Consistent with the results of the present study, previous studies have reported that the variant −842 C allele is significantly associated with a decreased risk of cancer (17,19,20,22–24). By contrast, no significant association between the −842 G>C SNP and cancer risk was identified in a study of hepatocellular carcinoma (19) and breast cancer by Naidu *et al* (21). Furthermore, previous functional analyses of the *PIN1* promoter polymorphism (−842 G>C) have demonstrated that *PIN1* gene expression, induced by the −842 C allele, was significantly lower than those induced by the variant −842 G allele via inhibition of the transcriptional activity of *PIN1* (17,22,24). In addition, Lu *et al* (17) indicated that the −842 CC genotype was associated with lower levels of *PIN1* protein expression in lung cancer samples. Therefore, the −842 C variant genotype may lead to a reduced expression of PIN1 and may be associated with a reduced risk of cancer. Functional studies of the *PIN1* promoter polymorphism (−842 G>C) further support the results of the present study, whereby individuals with variant GC and GC/CC genotypes were significantly associated with a decreased risk of cancer.

Accounting for the tumor origin may affect the results of the meta-analysis, therefore, stratified analyses were performed according to the cancer type. With the exception of the ‘other cancers’ subgroup, it was found that the C allele may be a protective factor against breast and lung cancer. Although the reasons for these discrepancies are not completely understood, one factor that may contribute to the discrepancies between the various studies is that this polymorphism may exert a particular effect at different cancer sites. Additionally, even at the same tumor site, due to the potentially marginal effect of this genetic polymorphism on cancer risk and the relatively small sample size of certain studies, the discrepancies may become apparent. For example, Han *et al* (20) found the *PIN1* promoter polymorphism (−842 G>C) was associated with a decreased risk of breast cancer, however, Naidu *et al* (21) did not come to this conclusion. With regard to other types of cancer in the present analysis, just one study was available for each specific cancer site, which may have limited the identification of a reliable association. For lung cancer, only two studies were included in the analysis. Although, breast cancer was investigated in four studies, each study had a small sample size. Therefore, the results of the present study must be interpreted with caution and large-scale, detailed and mechanistic studies are required to support the association between the *PIN1* promoter polymorphism (−842 G>C) and decreased cancer risk.

In the subgroup analysis of ethnicity, a decreased risk in C allele carriers was identified among Asian individuals, although not in European individuals (Table II). Although the exact mechanism for this ethnic discrepancy is unclear, certain factors may account for it. A possible reason is variations in genetic backgrounds, and/or environmental and social factors in the different populations. In addition, the different C allele frequency may be a reflection of natural selection stresses or indicate that the C allele together with other unidentified genes are involved in cancer development. Other factors, including selection bias, different matching criteria, misclassifications on disease status and publication bias may also be involved. Furthermore, only three studies included European individuals, therefore, larger-scale studies and combined analysis are required to further support the association between the *PIN1* promoter polymorphism (−842 G>C) and decreased cancer risk.

The test of heterogeneity markedly influenced the results of the present meta-analysis. Evident heterogeneity between the studies was observed in the overall comparisons. Thus, the studies were stratified by cancer type, ethnicity and sample size. It was found that the sources of heterogeneity were the cancer site and ethnicity, indicating that ethnicity and cancer type are significant when an individual possesses the same polymorphisms as another individual.

When interpreting the results of the current study, certain limitations of the meta-analysis must be considered. Firstly, the results were based on unadjusted estimates, however, a more precise analysis is required when more detailed, individual data becomes available, which would allow for an adjusted estimate including other factors, such as age and gender. Furthermore, a lack of information for the data analysis may result in confounding bias. Secondly, for a number of the reviewed studies the original data was not available, which limited further evaluation of potential interactions, as the interactions among gene-gene, gene-environment and different polymorphic loci of the same gene, may modulate cancer risk. Thirdly, in the present meta-analysis, the studies were all based on hospital patients and only contained Asian and European individuals. Thus, validations with larger population-based studies in different ethnic groups are required. Finally, the number of published studies was not sufficiently large for a comprehensive analysis, particularly for a specific cancer type. However, this meta-analysis also had certain advantages. Firstly, a substantial number of cases and controls were pooled from different studies, which significantly increased the statistical impact of the analysis. Secondly, the quality of the case-control studies that were included in current meta-analysis were satisfactory and no publication bias was detected, indicating that the entire pooled result was unbiased.

In conclusion, the present meta-analysis provides clear evidence that the *PIN1* promoter polymorphism (−842 G>C) contributes to a decreased cancer risk, supporting the hypothesis that the polymorphism may present as a biomarker for susceptibility to cancer. However, large studies investigating different ethnic groups using standardized unbiased methods, involving specifically selected cancer patients and well-matched controls, with more detailed individual data are required to validate the results of the current study. Finally, investigations of the gene-environment interaction may lead to an improved, more comprehensive understanding of the roles of *PIN1* polymorphisms in the etiology of cancer.

## Figures and Tables

**Table I:** Features of the studies included in the present meta-analysis.

First author (year)	Tumor type	Country	Patient ethnicity	Source of control group	Genotyping method	HWE	Sample size (case/control)
Segat (2007)	Liver cancer	Italy	European	Hospital	PCR-RFLP	NA	228/250
Lu (2011)^a^	Lung cancer	China	Asian	Hospital	PCR-RFLP	>0.05	1056/1056
Lu (2011)^b^	Lung cancer	China	Asian	Hospital	PCR-RFLP	>0.05	503/623
Naidu (2011)	Breast cancer	Malaysia	Asian	Hospital	PCR-RFLP	0.831	107/80
	Breast cancer	China	Asian	Hospital	PCR-RFLP	0.503	219/111
	Breast cancer	India	Asian	Hospital	PCR-RFLP	0.901	61/61
Han (2010)	Breast cancer	USA	European	Hospital	PCR-RFLP	0.160	467/488
Lu (2009)	SCCHN	USA	European	Hospital	PCR-RFLP	0.640	1006/1007
Lu (2012)	Nasopharyngeal cancer	China	Asian	Hospital	PCR-RFLP	0.063	178/156
You (2013)	Esophageal cancer	China	Asian	Hospital	PCR-RFLP	0.312	699/729

a Samples collected between March 2007 and 2009 in Southern China;

b Samples collected between March 2008 and May 2010 in Eastern China.

PCR, polymerase chain reaction; RFLP, restriction fragment length polymorphism; SCCHN, squamous cell carcinoma of the head and neck; NA, not available; HWE Hardy-Weinberg equilibrium.

**Table II:** Summary OR of the *PIN1* (−842G>C) polymorphism with regard to cancer risk.

			CC vs. GG		GC vs. GG		GC/CC vs. GG (dominant)		CC vs. GC/GG (recessive)	
						
Variable	n^b^	Cases/Controls	OR (95% CI)^c^	P-value^d^	OR (95% CI)^c^	P-value^d^	OR (95% CI)^c^	P-value^d^	OR (95% CI)^c^	P-value^d^
Cancer type										
Breast	4	854/740	0.53 (0.26–1.11)	0.498	0.72 (0.57–0.90)	0.408	0.71 (0.56–0.88)	0.493	0.58 (0.28–1.20)	0.483
Lung	2	1559/1679	0.77 (0.33–1.84)	0.721	0.64 (0.52–0.79)	0.814	0.65 (0.53–0.79)	0.762	0.82 (0.35–1.95)	0.713
Other	4	2111/2142	0.78 (0.33–1.85)	0.066	0.80 (0.52–1.25)	0.001	0.85 (0.60–1.20)	0.006	0.81 (0.31–2.07)	0.034
Ethnicity										
Europen	3	1701/1745	0.70 (0.38–1.26)	0.627	0.93 (0.57–1.50)	0.001	0.89 (0.59–1.35)	0.004	0.73 (0.40–1.31)	0.483
Asian	7	2823/2816	0.83 (0.52–1.33)	0.126	0.66 (0.57–0.76)	0.564	0.67 (0.58–0.77)	0.777	1.05 (0.64–1.74)	0.105
Sample size^a^										
≤500	6	1260/1146	0.66 (0.29–1.49)	0.091	0.85 (0.56–1.29)	0.002	0.86 (0.63–1.19)	0.022	0.68 (0.28–1.62)	0.053
>500	4	3264/3415	0.67 (0.38–1.17)	0.771	0.68 (0.59–0.78)	0.786	0.68 (0.59–0.78)	0.738	0.82 (0.44–1.52)	0.934
Total	10	4524/4561	0.78 (0.54–1.12)^e^	0.273^e^	0.75 (0.62–0.90)^e^	0.008^e^	0.75 (0.64–0.88)^e^	0.046^e^	0.84 (0.58–1.20)^e^	0.181^e^

a Two cases and controls.

b Number of comparisons.

c Random-effects model was used when P=0.05 for the heterogeneity test, otherwise the fix-effects model was used.

d Q-test for heterogeneity.

e Mean values.

OR, odds ratio; CI, confidence interval.
